# Mesenchymal Stem-Cell Derived Exosome Therapy as a Potential Future Approach for Treatment of Male Infertility Caused by *Chlamydia* Infection

**DOI:** 10.3389/fmicb.2021.785622

**Published:** 2022-01-13

**Authors:** Mahin Izadi, Laleh Dehghan Marvast, Mohammad Ebrahim Rezvani, Marzieh Zohrabi, Ali Aliabadi, Seyed Alireza Mousavi, Behrouz Aflatoonian

**Affiliations:** ^1^Research and Clinical Center for Infertility, Yazd Reproductive Sciences Institute, Shahid Sadoughi University of Medical Sciences, Yazd, Iran; ^2^Department of Reproductive Biology, School of Medicine, Shahid Sadoughi University of Medical Sciences, Yazd, Iran; ^3^Andrology Research Center, Yazd Reproductive Sciences Institute, Shahid Sadoughi University of Medical Sciences, Yazd, Iran; ^4^Department of Physiology, School of Medicine, Shahid Sadoughi University of Medical Sciences, Yazd, Iran; ^5^Infectious Disease Research Center, Shahid Sadoughi University of Medical Sciences, Yazd, Iran; ^6^Stem Cell Biology Research Center, Yazd Reproductive Sciences Institute, Shahid Sadoughi University of Medical Sciences, Yazd, Iran; ^7^Department of Advanced Medical Sciences and Technologies, School of Paramedicine, Shahid Sadoughi University of Medical Sciences, Yazd, Iran

**Keywords:** exosomes, mesenchymal stem cells, *Chlamydia trachomatis*, infectious diseases, male infertility

## Abstract

Some microbial sexually transmitted infections (STIs) have adverse effects on the reproductive tract, sperm function, and male fertility. Given that STIs are often asymptomatic and cause major complications such as urogenital inflammation, fibrosis, and scarring, optimal treatments should be performed to prevent the noxious effect of STIs on male fertility. Among STIs, *Chlamydia trachomatis* is the most common asymptomatic preventable bacterial STI. *C. trachomatis* can affect both sperm and the male reproductive tract. Recently, mesenchymal stem cells (MSCs) derived exosomes have been considered as a new therapeutic medicine due to their immunomodulatory, anti-inflammatory, anti-oxidant, and regenerative effects without consequences through the stem cell transplantation based therapies. Inflammation of the genital tract and sperm dysfunction are the consequences of the microbial infections, especially *Chlamydia trachomatis*. Exosome therapy as a noninvasive approach has shown promising results on the ability to regenerate the damaged sperm and treating asthenozoospermia. Recent experimental methods may be helpful in the novel treatments of male infertility. Thus, it is demonstrated that exosomes play an important role in preventing the consequences of infection, and thereby preventing inflammation, reducing cell damage, inhibiting fibrogenesis, and reducing scar formation. This review aimed to overview the studies about the potential therapeutic roles of MSCs-derived exosomes on sperm abnormalities and male infertility caused by STIs.

## Introduction

A prominent etiological factor in male infertility is genital tract infection. The infertility may be induced by various mechanisms, such as damage to gametogenic cells, decrease in the quality of sperm, and obstruction of the male reproductive tract (Keck et al., 1998; Sanocka-Maciejewska et al., 2005). The most common sexually transmitted microorganisms are *Chlamydia trachomatis* (*C. trachomatis*) (Nieschlag et al., 1997; Keck et al., 1998; Ombelet et al., 2008). There are controversial opinions on the role of *C. trachomatis* in male infertility (Dehghan Marvast et al., 2017). Several studies have shown that male infertility induced by chlamydial infection occurs in different forms of sperm abnormalities such as loss of mitochondrial membrane potential, increase in apoptosis through the activation of caspase 3 (Sellami et al., 2014) and DNA damage (Dehghan Marvast et al., 2018), and changes in sperm quality (Bezold et al., 2007; Sellami et al., 2011, 2014). Also, other studies have claimed that this microorganism infection causes an inflammatory reaction which leads to seminal tubes occlusion (Dohle, 2003; Dehghan Marvast et al., 2016; Zhou et al., 2021). Many sexually transmitted infections (STIs) pathogens such as *C. trachomatis* are asymptomatic in subfertile men (Sharma and Agarwal, 1996; Bezold et al., 2007; Geisler, 2010; Hakimi et al., 2014; Bai et al., 2021). Screening and treatment should be performed to prevent the detrimental effect of *C. trachomatis* on male fertility (Geisler, 2010; Bryan et al., 2019). Widespread antibiotics are currently the most common treatment for chlamydial infection (Murray and McKay, 2021), and this treatment can effectively alleviate the infection and ameliorate sperm quality (Gallegos et al., 2008; Hamazah and Al-Dahmoshi, 2021). However, antibiotic resistance is one of the remaining challenges for this treatment, especially in patients with multidrug resistance (Hamazah and Al-Dahmoshi, 2021; Vanić et al., 2021).

The new experimental methods of the infertility treatment are stem cell and exosome applications. Because of the limitations using live cells injections and also the therapeutic effect of their paracrine substances (Janockova et al., 2021), MSC–derived exosomes containing bioactive molecules have been recently used in studies of infertility treatment. Exosome therapy as a noninvasive approach has shown promising results on the ability to regenerate damaged sperms and treating asthenozoospermia by their repairing molecules and counteracting with the reactive oxygen species (ROS) (Kharazi and Badalzadeh, 2020). These experimental methods may be helpful in the novel treatments of male infertility. This review aimed to overview the studies about the therapeutic potentials of the MSCs-derived exosomes on sperm abnormalities and male infertility caused by STIs.

## *C. trachomatis*: Cell Biology

*Chlamydia* is a gram negative bacterium, an obligate intracellular parasite, divided into 18 serovars (A-C, D-K, and L1-L3) distinguished by the antigen named the Major Outer Membrane Protein. This antigen gives the pathologic properties to the serovars D-K and may play an essential role in genital tract infection (Murray and McKay, 2021). Unlike other microorganisms, *C. trachomatis* has two distinct developmental cycles, the infectious type or elementary body (EB) and intracellular replicative type or reticulate body (RB). Both types of this bacterium are metabolically active, although their energy sources are different (Omsland et al., 2012). Expressions of different antigens during the cell cycle lead to difficulties in eradicating the bacterium (Paavonen and Eggert-Kruse, 1999; Murray and McKay, 2021). EB form attaches to the host cell and enters it and protects itself from host cellular defense by formation of vacuoles and inclusions (Hosseinzadeh et al., 2000).

## Pathophysiological Mechanisms

Approximately 50% of *C. trachomatis* infections in men are asymptomatic, but it can cause epididymitis, epidiymo-orchitis, urethritis, and prostato-vesiculitis (Eley et al., 2005; Rana et al., 2016). Because of wide range of pathological changes and tissue injuries in the urogenital tract, it is necessary to briefly review the pathophysiology of *C. trachomatis*. This bacterium first attaches to the epithelial cells in the urogenital tract, and this is where immunological reactions are initiated. The infected non-immune cells recognize different invaded pathogens such as *C. trachomatis* by their PRRs (pathogen recognition receptors) (Mackern-Oberti et al., 2013). The interaction between non-immune host cell and bacterium leads to secretion of many cytokines (IL-1, IL-8, IL-6) (Al-mously and Eley, 2007; Redgrove and McLaughlin, 2014) and tumor necrosis factor alpha (TNFα); these, in turn, recruit natural killer (NK) cells, DCs, neutrophils, macrophage, T cells, and B cells (Redgrove and McLaughlin, 2014). One of the most substantial cellular immune reactions against chlamydia infection is mediated by antigen-specific IFN-γ secreting CD4^+^, CD8^+^ T cells, and NK cells. Also, elimination of chlamydial infection depends on IFN-γ secreting CD4^+^ Th1 cells (Cain and Rank, 1995; Perry et al., 1997). Immune cells also generate chronic inflammation by increasing the production of ROS and releasing molecules with degradative properties including defensins, elastase, collagenase, cathespins, and lysozyme. Finally, the immune reactions lead to tissue remodeling and scarring in the reproductive system (Redgrove and McLaughlin, 2014).

## Effects of *C. trachomatis* on Sperm and Male Infertility

Infertility in men is caused by various reasons such as genetic abnormalities, testicular damage, varicocele, immunological subjects, systemic diseases, environmental factors, endocrine disorders, and exposure to gonadotoxic agents (Dohle et al., 2004; Jungwirth et al., 2012). In addition to the above-mentioned factors, male genital tract infection and inflammation play a devastating role in 8–35% of male infertility. Infectious factors such as fungi, parasites, viruses, and several other microorganisms including *C. trachomatis*, *Neisseria gonorrhoeae*, *Ureaplasma urealyticum*, and *Trichomonas vaginalis* are involved in these disorders, which can affect the testis, epididymis, accessory sex glands, sperm cell function, and finally fertility (Isaiah et al., 2011). The most common cause is *C. trachomatis*, which leads to infertility by affecting both the sperm and the male reproductive tract (Nieschlag et al., 1997; Keck et al., 1998; Ombelet et al., 2008).

Some studies have regarded the relationship between *C. trachomatis* infection and semen quality. Semen of *C. trachomatis* infected patient indicates reduced volume, decrease in sperm motility, change in sperm concentration, and pH alteration (Veznik et al., 2004; Rana et al., 2016). It seems that aforementioned effects on the sperm can be due to Chlamydia lipopolysaccharide (LPS) which interacts with CD14 on the sperm membrane and leads to elevating production of ROS and eventually induced apoptosis (Harris et al., 2001). Another study demonstrated that *C. trachomatis* infection can cause rising in the mitochondria membrane potential, caspase 3 activation, and finally apoptosis induction in spermatozoa (Sellami et al., 2014). Moreover, externalization of phosphatidylserine (PS) in sperm membrane and DNA fragmentation has been reported as a negative impact of *C. trachomatis* on sperm function and fertility (Satta et al., 2006). In addition, several studies have reported infections of the reproductive system can cause leukocytospermia, and the leukocytes are able to produce oxidative damage of the sperm plasma membrane and DNA through the release of cytokines, free oxygen radicals, and reactive nitrogen (Anderson and Hill, 1988; Aitken and West, 1990; Hamada et al., 2011).

## Current Treatment

Current treatment includes azithromycin 1 g single dose or doxycycline 100 mg orally twice daily for 7 days (Stamm et al., 1995; Dieterle, 2008; Mishori et al., 2012). Timely management of sexual intercourse and sex partner treatment are also necessary to reduce the re-infection risk (Centers for Disease Control and Prevention, 1998a,b; Workowski and Berman, 2011). Approximately 50% of *C. trachomatis* infections in men are asymptomatic and can cause many complications (Pacey and Eley, 2004; Eley et al., 2005; Rana et al., 2016). Thus, screening programs are necessary to prevent long-term complications of *C. trachomatis* infection such as epididymitis, accessory sex glands inflammation, testicular atrophy, tubular tract occlusion, and male infertility (Paavonen and Eggert-Kruse, 1999). While treatment with antibiotics significantly clears sexually transmitted patients, this treatment has its limitations (Kong et al., 2014). First, screening programs to identify chlamydia infected individuals are costly and impractical, so they are limited to symptomatic patients who are following their diseases (World Health Organization, 2016). Antibiotic therapy may also impair the production of a sustained protective immune response to chlamydia (Patton et al., 2014).

Vaccines have long been designed to treat chlamydia infection. Despite numerous successes in this field, there are still issues that have limited human access to deliver effective vaccines without complication. Biological characteristics, two-phase life cycle, and especially the ability of this bacterium to hide from the view of the immune system are the main reasons for this limitation in vaccine production. Providing a reliable and effective vaccine for *Chlamydia* prophylaxis is still awaiting further research and possibly shifting from whole-cell based vaccines to subunit-based vaccines, especially considering the role of MOMP (Murray and McKay, 2021).

Importantly, in some cases in which the complications still remained following antibiotic therapy, a new therapeutic approach is necessary for treatment. In this regard, MSCs-derived exosomes have been shown to have critical roles such as anti-inflammatory, antioxidant, regenerative and fibrogenesis inhibiting, and wound and fracture healing (Janockova et al., 2021), which can be considered a novel approach in the male infertility complications of *C. trachomatis* infection.

## Exosome: General Aspects

In different multicellular organisms, the intercellular communication occurs through cell-to-cell contact or through the secretion of molecules (Lai, 2004). Two decades ago, another mechanism was considered in the intercellular communication, which involves the transfer of extracellular vesicles that release from the plasma membrane into the intercellular space under physiological and pathological events and influence the other cells in paracrine and endocrine manners (György et al., 2011). Based on biosynthesis pathways and their size, the extracellular vesicles are divided into three categories: micro vesicles (50–3,000 nm), exosomes (40–100 nm), and apoptotic bodies (800–5,000 nm) (Yamamoto et al., 2016). Other studies have also mentioned other sizes for exosome: (30–100) (Wang et al., 2017), (50–150 nm) (Théry et al., 2018), (40–160 nm) (Kalluri, 2016), and (50–100 nm) (Gould and Raposo, 2013). Recently exosomes have attracted huge attention from researchers due to their genetic material and protein shuttling ability to other cells with various contents according to their origin (Han et al., 2016). Exosomes secrete from T cells (Nolte-‘t Hoen et al., 2009), B cells (Clayton et al., 2005), macrophages (Bhatnagar et al., 2007), epithelial cells (Skogberg et al., 2015), endothelial cells (Song et al., 2014, 2015), as well as MSCs (Yeo et al., 2013). The vesicles with exosomal characteristics have been also founded in the various body fluids such as semen (Fabiani et al., 1994; Arienti et al., 1999; Park et al., 2011; Aalberts et al., 2012), blood (Blanc et al., 2005; Caby et al., 2005; Yunusova et al., 2016), breast milk (Admyre et al., 2007; Qin et al., 2016; Miyake et al., 2020), ascites fluid (Andre et al., 2002; Navabi et al., 2005; Runz et al., 2007), saliva (Ogawa et al., 2008; Michael et al., 2010), amniotic fluid (Asea et al., 2008; Zhang et al., 2021), urine (Gonzales et al., 2010; Street et al., 2017), and bile (Masyuk et al., 2010; Sagredo et al., 2017). Because exosomes are in nano sized range, they spread through body fluids and easily penetrate through tissues and affect targeted cells (Phinney and Pittenger, 2017), even if those cells are far away (François et al., 2006). The synthesis, secretion, and effects of the extracellular vesicles were intensively considered in the past few decades so that it led to the creation a scientific association named the International Society for Extracellular Vesicles (ISEV) (Kowal et al., 2014). Various techniques for isolation and detection of exosomes have been reported in recent studies. Isolation techniques include differential ultracentrifugation (Parolini et al., 2009), density gradient (Beyer and Pisetsky, 2010), size exclusion chromatography (Livshits et al., 2015), ultrafiltration (Greening et al., 2015), immunological separation (Beyer and Pisetsky, 2010), isolation by sieving (Taylor and Shah, 2015), cell sorting (Peterson et al., 2015), polymer-based precipitation (Grant et al., 2011), and microfluidic technologies (Oves et al., 2018). Exosome identification techniques include electron microscopy, western blot, flow cytometry, and nanosight tracking analysis (Crenshaw et al., 2018). The latest methods and techniques are RNA-seq techniques (Jeppesen et al., 2019).

## Exosome Biogenesis

Exosome generation, which was conserved during evolution, is a continuation of the extracellular ligands internalization and endocytosis process, which is carried out by the curvature of the plasma membrane and budding inside the intracellular endosome that leads to the formation of multivesicular bodies (MVB). Later, the MVB, which contains intraluminal vesicles (ILVs) that can be the precursors of the exosome, either leads to fusion with lysosomes and degradation, or undergoes exocytic merging with plasma membranes and exosome secretion (Stoorvogel et al., 2002; Février and Raposo, 2004; Colombo et al., 2014; Kowal et al., 2014; Meldolesi, 2018; Xunian and Kalluri, 2020). Molecular mechanisms of ILV generation depend firstly on the endosomal sorting complex required for transport (ESCRT), a molecular apparatus comprised of four sets including ESCRT-0 which consists of two subunits HRS (hepatocyte growth factor-regulated tyrosine kinase substrate) and STAM1/2 (signal transducing adaptor molecule1/2) (for cargo clustering and sorting), ESCRT-I and ESCRT-II (induce membrane curvature and vesicle budding), and ESCRT-III (membrane deformation and vesicle detachment) (Henne et al., 2011; Meldolesi, 2018; Xunian and Kalluri, 2020). The subordinate proteins (Vps4-Vta1 complex, Tsg101, Vps24, Vps37, Vps2, and Alix) are also critical for exosome biogenesis pathway (Henne et al., 2011). ESRT apparatus is also involved in the deubiquitination of some proteins that are ubiquitinated in ILVs (Henne et al., 2011; Meldolesi, 2018). The deubiquitination is mediated by the protein tyrosine phosphatase HD-PTP, which is an essential process for exosome function (Meldolesi, 2018). The subordinate proteins (class I AAA ATPase Vps4) can cause the ESCRT apparatus recycling (Xunian and Kalluri, 2020). In addition to the ESRT pathway, there are other independent pathways, for example, ceramide derived from sphingomyelin can cause membrane deformation and vesicles budding within the MVB (Trajkovic et al., 2008; Henne et al., 2011).

## Exosome Composition

Exosomes are extra cellular vesicles that are secreted from different cells under both normal and disease conditions and represent cells function or even as diagnostic markers of diseases. Existence of mRNA and miRNA within the exosomes has led to more studies in recent years, making this field more attractive (Valadi et al., 2007). The exosomes carry bimolecular content such as protein (membrane proteins, cytosolic and nuclear proteins, and extracellular matrix proteins), lipid, and nucleic acid which are different between cells (McAndrews and Kalluri, 2019). This content can be verified and accessed in the Exocarta,^1^ a manually curated web-based database. The current Exocarta is based on about 286 studies on exosomes and contains about 41,860 proteins, 1,116 lipid, and more than 7,540 RNAs from 10 various species (Keerthikumar et al., 2016). Several most common proteins on the exosomal surface such as tetraspanins (CD63, CD81, CD82, and CD9) are known as membrane scaffolds (Ma et al., 2020); in addition to the above-mentioned tetraspanins, in the MSC-derived exosomes, there are expressions of CD73, CD44, and CD90 (Ramos et al., 2016). Exosomes present antigen proteins such as major histocompatibility complex (MHC) I and II, flotillin-1, and integrins. Other proteins include MVB biogenic proteins such as ESCRT complex 0,-1,-II,-III, Alix, syntenin, TSG101, membrane transporters, and fusion proteins such as RAB protein, RAP1B, RhoGDIs and annexins (Ma et al., 2020), several enzymes such as glyceraldehydes- 3-phosphate dehydrogenase (GAPDH), phosphoglycerate kinase 1 (PGK1) (Van Niel et al., 2011; Charrin et al., 2014), and alanylaminopeptidase N (Ma et al., 2020), a number of chaperones such as heat shock protein 70 (HSP70), heat shock cognate 70 (HSC70) (Van Niel et al., 2011; Charrin et al., 2014), HSP90, HSP60, and HSP8 (Ma et al., 2020), adhesion proteins such as L1 cell adhesion molecule (L1CAM), and lysosomal associated membrane protein 2 (LAMP2) (Urbanelli et al., 2013).

Exosomes are also rich in genetic materials. Different types of RNAs including mRNAs and miRNAs, vault RNAs (vtRNAs), Y-RNAs, ribosomal RNAs (rRNAs), and transfer RNA (tRNAs) (Squadrito et al., 2014; Vojtech et al., 2014; Shurtleff et al., 2017). Also, various types of DNAs in exosomes are double-stranded DNAs (dsDNA) (Thakur et al., 2014), mitochondrial DNAs (mtDNAs) (Guescini et al., 2010), and single-stranded DNAs (ssDNAs) (Balaj et al., 2011).

Other exosome contents are lipid compositions including cholesterol, phosphatidylserine (PS), sphingomyelin, ceramide, lysobisphosphatidicacid, and phosphatidylethanolamine (PE), which play an important role in membrane structure and exosome formation and are secreted in the extracellular environment (Skotland et al., 2019).

Exosomes with lipid bilayer membrane can protect genetic material and other contents through transportation to the targeted cell (Fu et al., 2019). MSC-derived exosomes transmit their composition to the targeted cells either *via* plasma membrane fusion or membrane receptor function which lead to the exosome internalization (Harrell et al., 2019a).

## Mesenchymal Stem Cells-Derived Exosomes

MSCs which are mainly tissue specific stem cells can be isolated from adult (Akyash et al., 2020) and fetal (Hoseini et al., 2020) sources. MSCs can be also be produced from pluripotent human embryonic stem cells (hESCs) (Javidpou et al., 2021). Different cells secrete exosomes that have similar protein molecules and biological activities. Immune modulation, regeneration, tissue repair, and promotion of angiogenesis are the similar *in vivo* and *in vitro* therapeutic effects of MSC-derived exosomes. These similar activities may be related to the presence of common protein signature in all MSCs-derived exosomes (van Balkom et al., 2019). However, MSCs are a massive source for production of exosomes, more accessible, and highly proliferative (Cheng et al., 2017) and that makes them more suitable for different fields of research. Moreover, exosomes derived from specific types of MSCs have unique properties (Tang et al., 2021). Additionally, different specific cells secrete exosome containing unique protein molecules and exert biological activity (Simpson et al., 2008). For example, in a recent study, amelioration of the spermatogonia injuries by Sertoli cell-derive exosome was revealed (Salek et al., 2021).

## Role of Mesenchymal Stem Cells-Derived Exosomes in Inflammation and Cellular Damage

Numerous studies have shown the potential of MSC-derived exosomes for treatment of diseases, which can be used as vaccines (prophylaxis), treatment, disease biomarkers, and drug delivery (Wang et al., 2017; Janockova et al., 2021).

It has been demonstrated that MSC-derived exosomes exhibit a crucial role in repair of the epithelium damage and re-epithelialization (Zhang et al., 2015a), angiogenesis (Shabbir et al., 2015; Zhang et al., 2015b), and prevention of the scar formation by suppressed myofibroblast differentiation (Fang et al., 2016). Studies have also reported that MSC-derived exosomes containing miRNAs can reduce inflammation by transforming the pro-inflammatory macrophage M1 to anti-inflammatory phenotype M2. The phenotype M2 reduces local interleukin-1β, interleukin-6, and tumor necrosis factor alpha (TNF-α) and increases the secretion of anti-inflammatory factors such as IL-10 as well as immune regulation (Wei et al., 2019; Zhao et al., 2019). Recent study demonstrated that MSC-derived exosomes can cause suppression of CD4^+^ Th1 and Th17 and induction of T regulatory cells (Treg) expansion which it in turn regulates and suppresses the immune system (Harrell et al., 2019b). Also, the protective effects of MSC-derived exosomes have been mediated *via* oxidative stress suppression and maintain balance of cellular redox state (Yang et al., 2015).

Studies have also shown the important role of MSC-derived exosomes in tissue repair after injury, the effect that is mediated by inducing cell differentiation, proliferation, and prevention of apoptosis. The miRNAs such as miR-21-5p, miR-144, and miR-19a are the factors that inhibit apoptosis in the MSC-derived exosomes and reduce apoptotic proteins such as caspase 3, caspase 8, and caspase 9 after tissue injury (Yu et al., 2015; Li et al., 2019; Wen et al., 2020).

**FIGURE 1:**
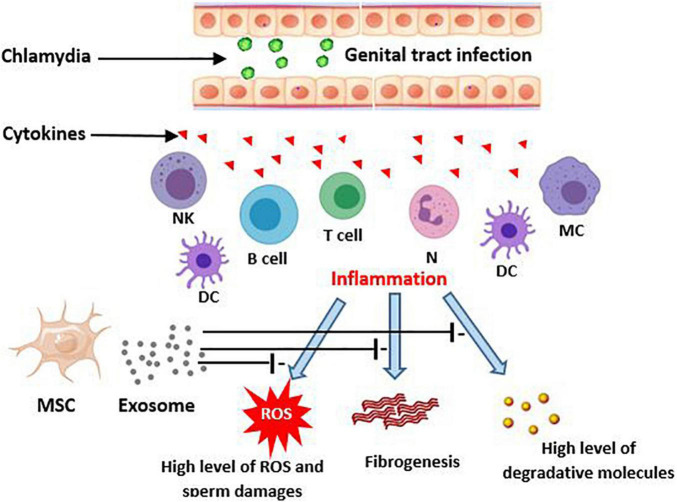
Potential effects of MSCs-derived exosomes on consequences of chlamydia infection in the genital tract. Genitalia tract infection with chlamydia evokes an inflammatory immune response by epithelial and local immune cells. This, in turn, produces the high level cytokins that initiate a more severe immune reaction. The responses may result in male genital inflammation and fibrosis. On the other hand, the inflamed tissue can lead to creation of ROS production and then sperm damages. MSC derived exosomes potentially improve these consequences of chlamydia induced inflammation. DC, Dendritic cells; MC, Macrophage; NK, Natural killer; MSC, Mesenchymal stem cell; ROS, Reactive oxygen species.

In the inflammatory response of colitis it has been reported that MSC-derived exosomes attenuate inflammation through decrease in TNF-α, nuclear factor kappaBp65 (NF-κBp65), cyclooxygenase-2 (COX-2), inducible nitric oxide synthase (iNOS), interleukin-1β (IL-1β), and increase in expression of IL-10. Alleviation of LPS-induced inflammation and acute respiratory distress syndrome (ARDS) by MSC-derived exosomes has been demonstrated (Deng et al., 2020). Another study on premature ovarian failure reported that MSC-derived exosomes with miR-644-5p can cause apoptosis inhibition *via* impressing p53 and recover normal function in ovarian granulosa cell (Sun et al., 2019). Considering the male infertility caused by *C. trachomatis* has inflammation-based pathology (Lotti and Maggi, 2013; Redgrove and McLaughlin, 2014), exosome therapy may be a beneficial technique to attenuate the cell injuries and the tissue remodeling such as occurrence of fibrosis and scar formation (Figure 1).

## Role of Mesenchymal Stem Cells-Derived Exosomes in Infection

The antimicrobial properties of MSC-derived exosomes have been reported by several clinical trials (Krasnodembskaya et al., 2010; Harman et al., 2017; Cortés-Araya et al., 2018). Studies also showed that exosomes contain antimicrobial peptides (AMPs) and the proteins that have bactericidal effect (Gläser et al., 2005; Krasnodembskaya et al., 2010; Allen and Stephens, 2011; Alcayaga-Miranda et al., 2017). MSC-derived exosomes indicated the therapeutic effect on lung injury that induced by *E. coli* (Zhu et al., 2014). Also, enhancing anti-microbial function of immune cells infiltration in lung by MSC-derived exosome has been reported in an animal study (Hao et al., 2019). A previous study revealed that exosomes can protect the brain against sepsis induced in an experimental model (Chang et al., 2018). MSC-derived exosomes enhanced the bacterial phagocytosis capability of the monocytes in severe bacterial pneumonia (Monsel et al., 2015) and enteric infections (Islam et al., 2001). Moreover, immunoregulatory properties of monocytes and decrease in inflammatory cytokine secretion were observed after use of the exosomes (Monsel et al., 2015). There is evidence that MSC-derived exosomes with their immunomodulatory, pro-angiogenesis, and anti-inflammatory activities can prevent inflammatory responses and alleviate COVID-19-induced pneumonia and lung injury (Raghav et al., 2021). In sum, these evidences about the role of exosomes in infections, especially their effects in increase of phagocytosis by monocytes, generate promising reasons to give them a potential property for eradication of the micro-organisms.

MSC-derived exosomes, as a natural carrier, possess a capability of embedding and delivering antibiotics and drugs. The use of exosomes as carriers leads to reduction of drugs that metabolize, targeted drug delivery, and thus overcome drug resistance (Bartolini et al., 2013; Yeo et al., 2013; Batrakova and Kim, 2015; Gao et al., 2018; Oves et al., 2018; Herrmann et al., 2021). However, exosome modifications change the functions and therapeutic effects of these vehicles (Ma et al., 2017; Tamura et al., 2017).

## Potential Therapeutic Role of Mesenchymal Stem Cells-Derived Exosome in Sperm Abnormality

**FIGURE 2:**
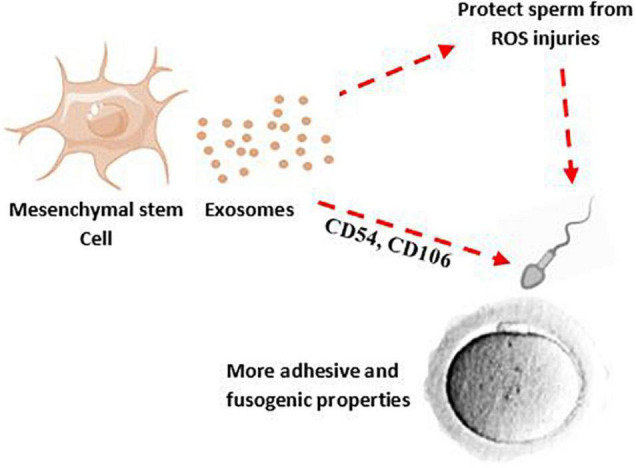
MSC-derived exosomes may decrease ROS production after chlamydia infection and their effects on sperm membrane and DNA. Therefore, MSCs-derived exosomes can potentially improve quality and adhesive properties of sperm.

To achieve proper male fertility, safe sperm manipulation is important. Recently, new methods such as the use of nanoparticles have been used to develop non-invasive techniques for treating and manipulating sperm (Feugang, 2017). The effectiveness and non-invasiveness of the nanoparticles such as exosome for mammalian sperm have been proven (Vilanova-Perez et al., 2020). According to animal studies, exosomes appear to be a promising avenue to restore spermatogenesis and sperm regeneration; a study has shown that amniotic fluid-derived exosome can restore sperm parameters such as motility, concentration, as well as the number of spermatogonia, spermatocytes, and ultimately male fertility (Mobarak et al., 2021). The protective effect of exosomes against sperm cryoinjuries (such as cell membrane injury, DNA damage) and oxidative stress produced by cryopreservation process and improvement of the post-thaw sperm parameters has been reported (Qamar et al., 2019; Mahiddine et al., 2020). Interestingly, treatment of spermatozoa with MSC-derived exosomes, in addition to improving sperm parameters after frozen-thawed, can increase sperm adhesive and fusogenic properties by adhesion molecules shuttling such as CD44, CD29, CD54, and CD106 (Mokarizadeh et al., 2013; Figure 2).

Exosomes contain different molecules such as RNAs that can be incorporated into immune or host cells. RNA sequencing analysis showed that microRNAs were the most frequent in exosomes (Huang et al., 2013). MSC-exosomes can play a role in injury repair and preventing apoptosis after injury through the miRNAs (e.g., miR-19a, miR-144, and miR-21-5p). The potential role of the miRNAs in improvement of chlamydial-induced sperm damages may confer a therapeutic application to the exosome. In addition, there are several clinical trials that demonstrated loading of exosomes with drugs or bioactive molecules (NCT01294072, NCT03608631, NCT01159288) for therapeutic proposes (NCT04602442, NCT04213248, NCT03437759, NCT04276987) (Herrmann et al., 2021). Therefore, it seems that exosomes can be used for treatment of sperm damage.

## Conclusion

There are reported evidences demonstrated regenerative, anti-microbial, and anti-inflammatory and anti-oxidant activities of exosomes. It is worthwhile to investigate and challenge the identity and effectiveness of the exosomes in the treatment and control of the consequences of male genitalia tract infections, especially chlamydia. MSC-derived exosomes therapy can lend itself as the potential treatment of male infertility caused by microbial infections in the near future.

## Author Contributions

MI: study design, investigation, and writing original draft. LD: validation of data and revising the manuscript. MR and MZ: helping on writing the manuscript. SM: validation of data. AA: helping on writing the first draft of the manuscript. BA: supervisor, validation of data, and revising the final version of the manuscript. All authors contributed to the article and approved the submitted version.

## Conflict of Interest

The authors declare that the research was conducted in the absence of any commercial or financial relationships that could be construed as a potential conflict of interest.

## Publisher’s Note

All claims expressed in this article are solely those of the authors and do not necessarily represent those of their affiliated organizations, or those of the publisher, the editors and the reviewers. Any product that may be evaluated in this article, or claim that may be made by its manufacturer, is not guaranteed or endorsed by the publisher.

## Footnotes

1
http://www.exocarta.org
